# Detection of Major Mutations in *CFTR, SERPINA1, HFE* Genes in Benign Unconjugated Hyperbilirubinemia Phenotype

**DOI:** 10.17691/stm2024.16.4.04

**Published:** 2024-08-30

**Authors:** A.A. Ivanova, N.E. Apartseva, A.P. Kashirina, E.G. Nemtsova, Y.V. Ivanova, M.V. Kruchinina, S.A. Kurilovich, V.N. Maksimov

**Affiliations:** MD, PhD, Senior Researcher, Laboratory of Molecular Genetic Testing of Therapeutic Diseases; Institution of Internal and Preventive Medicine — Branch of the Federal Research Center Institute of Cytology and Genetics, Siberian Branch of the Russian Academy of Sciences, 175/1 B. Bogatkova St., Novosibirsk, 630089, Russia; PhD Student, Junior Researcher, Laboratory of Genetic and Environmental Determinants of Human Life Cycle; Institution of Internal and Preventive Medicine — Branch of the Federal Research Center Institute of Cytology and Genetics, Siberian Branch of the Russian Academy of Sciences, 175/1 B. Bogatkova St., Novosibirsk, 630089, Russia; PhD Student, Junior Researcher, Laboratory of Genetic and Environmental Determinants of Human Life Cycle; Institution of Internal and Preventive Medicine — Branch of the Federal Research Center Institute of Cytology and Genetics, Siberian Branch of the Russian Academy of Sciences, 175/1 B. Bogatkova St., Novosibirsk, 630089, Russia; MD, PhD, Associate Professor, Department of Propaedeutics of Internal Diseases, Gastroenterology, and Dietology named after S.M. Ryssa; North-Western State Medical University named after I.I. Mechnikov, 41 Kirochnaya St., Saint Petersburg, 191015, Russia; Resident; Institution of Internal and Preventive Medicine — Branch of the Federal Research Center Institute of Cytology and Genetics, Siberian Branch of the Russian Academy of Sciences, 175/1 B. Bogatkova St., Novosibirsk, 630089, Russia; MD, DSc, Associate Professor, Leading Researcher, Gastroenterology Laboratory; Institution of Internal and Preventive Medicine — Branch of the Federal Research Center Institute of Cytology and Genetics, Siberian Branch of the Russian Academy of Sciences, 175/1 B. Bogatkova St., Novosibirsk, 630089, Russia; MD, DSc, Professor, Head of Gastroenterology Laboratory; Institution of Internal and Preventive Medicine — Branch of the Federal Research Center Institute of Cytology and Genetics, Siberian Branch of the Russian Academy of Sciences, 175/1 B. Bogatkova St., Novosibirsk, 630089, Russia; MD, DSc, Professor, Chief Researcher, Laboratory of Molecular Genetic Testing of Therapeutic Diseases; Institution of Internal and Preventive Medicine — Branch of the Federal Research Center Institute of Cytology and Genetics, Siberian Branch of the Russian Academy of Sciences, 175/1 B. Bogatkova St., Novosibirsk, 630089, Russia

**Keywords:** Gilbert’s syndrome, UGT1A1, HFE, CFTR, SERPINA1, nonconjugated hyperbilirubinemia

## Abstract

**The aim of the study** was to search for the associations of benign unconjugated hyperbilirubinemia phenotype with rs1799945 (H63D), rs1800562 (C282Y), rs1800730 (S65C) mutations of *HFE* gene, rs113993960 (ΔF508) of *CFTR* gene, rs28929474 (PIZ), rs17580 (PIS) mutations of *SERPINA1* gene.

**Material and Methods:**

The study design is case-control. The group with Gilbert’s syndrome (GS) phenotype (n=414; mean age — 36.7±15.9 years; 49.8% men) was formed by gastroenterologists, and included the individuals with unconjugated hyperbilirubinemia who underwent a standard clinical examination. The individuals with known causes of unconjugated hyperbilirubinemia were excluded from the group. The control group (n=429; mean age — 38.5±14.3 years; 52.2% men) was a random sampling from DNA banks of MONICA project participants, the screening of young people aged 25-44 and a one-time study of schoolchildren in Novosibirsk (Russia). DNA was isolated by phenol-chloroform extraction or by the express method (PROBA-RAPID-GENETIKA; DNA-Technology, Moscow, Russia) from venous blood. Genotyping of groups by nucleotide sequence rs1799945 (H63D), rs1800562 (C282Y), rs1800730 (S65C) of *HFE* gene, rs113993960 (ΔF508) of *CFTR* gene, rs28929474 (PIZ), rs17580 (PIS) of *SERPINA1* gene was performed by polymerase chain reaction followed by the analysis of fragment length polymorphism on a polyacrylamide gel.

**Results:**

According to the genotypes and alleles of the variants rs1799945 (H63D), rs1800562 (C282Y), rs1800730 (S65C) of *HFE* gene, rs113993960 (ΔF508) of *CFTR* gene, rs28929474 (PIZ), rs17580 (PIS) of *SERPINA1* gene, no statistically significant differences were found between the GS group and the control group (p>0.05).

**Conclusion:**

Nucleotide sequence variants rs1799945 (H63D), rs1800562 (C282Y), rs1800730 (S65C) of *HFE* gene, rs113993960 (ΔF508) of *CFTR* gene, rs28929474 (PIZ), rs17580 (PIS) of *SERPINA1* gene, or their combinations with rs3064744 of *UGT1A1* gene were found to have no association with GS.

## Introduction

The relevance of searching molecular genetic markers of benign unconjugated hyperbilirubinemia is beyond dispute, since the pathology is very common. Gilbert’s syndrome (GS) frequency in population is about 10%. GS is characterized by variable expressivity and penetrance that is evidence of the incompleteness of knowledge on the syndrome [1]. Insufficient correlation “phenotype–genotype” is one of significant criteria of the disease oligogeneity. Probably, for the European population except the frequent variant rs3064744 of *UGT1A1* gene, there are other nucleotide sequence variants of genes and molecular genetic mechanisms, which can explain the different degree of clinical symptoms of unconjugated hypebilirubinemia in different genotypes of *UGT1A1* gene variants.

As the evidence from clinical practice shows, the course of benign unconjugated hyperbilirubinemia can be influenced by the carriership of rare mutation alleles, which can cause other diseases (cystic fibrosis, alha-1-antitrypsin deficiency, hemochromatosis) affecting hepatic functions.

Cystic fibrosis development is related to *CFTR* gene mutations, which result in chloride canal function impairment controlling water and ion secretion and absorption in epithelial tissues. The most common mutation in *CFTR* gene in cystic fibrosis is the variant rs113993960 (ΔF508) of *CFTR* gene (OMIM 602421).

Type 1 hereditary hemochromatosis develops due to *HFE* gene mutations causing the dysfunction of the protein — an iron absorption regulator. Most cases of type 1 hereditary hemochromatosis are related to rs1799945 (H63D), rs1800562 (C282Y), rs1800730 (S65C) variants of *HFE* gene (OMIM 235200).

Alpha-1-antitrypsin deficiency is caused by *SERPINA1* gene mutations (most frequently — the variants rs28929474 (PIZ), rs17580 (PIS) of the gene) leading to the dysfunction of the encoding the protein genome — a serine protease inhibitor.

Such mutations cause serious impairments in hepatocytes functioning, it significantly decreasing their adaptability and compensability for any damage effects and factors, foremost, those caused by chemical and biological nature. For instance, there were reported cases of severe hepatic involvement in combination of rare *UGT1A1* gene variants with *HFE* gene mutations [2].

Thus, **the aim of the study** was to analyze the association of benign unconjugated hyperbilirubinemia with major mutations rs1799945 (H63D), rs1800562 (C282Y), rs1800730 (S65C) of *HFE* gene, rs113993960 (ΔF508) of *CFTR* gene, and rs28929474 (PIZ), rs17580 (PIS) of *SERPINA1* gene.

## Materials and Methods

The study design is case-control.

Gastroenterologists formed a group of patients with GS phenotype (n=414; mean age — 36.7±15.9 years; 49.8% men and 50.2% women). The group included the subjects with unconjugated hyperbiliubinemia, who had undergone a standard clinical examination. The individuals with known causes of unconjugated hyperbilirubinemia were excluded from the study. DNA was isolated by phenol-chloroform extraction or by the express method (PROBA-RAPID-GENETIKA; DNA-Technology, Russia) from venous blood.

The control group (n=429; mean age — 38.5±14.3 years; 52.2% men and 47.8% women) was a random sampling from DNA banks of MONICA (Multinational MONItoring of trends and determinants in CArdiovascular disease) project participants, the screening of young people aged 25-44 and a onetime study of schoolchildren in Novosibirsk (Russia). DNA was isolated by phenol-chloroform extraction from venous blood.

The study was carried put in accordance with Declaration of Helsinki (2013) principles, and approved by the Ethics Committee of Institution of Internal and Preventive Medicine — Branch of the Federal Research Center Institute of Cytology and Genetics, Siberian Branch of the Russian Academy of Sciences (Novosibirsk, Russia). All study participants gave their informed consent to a molecular genetic analysis.

The groups had no significant difference from each other by gender and age.

In both groups there were defined the frequencies of genotypes rs3064744 (the number of TA-repeats in a promoter) of *UGT1A1* gene by polymerase chain reaction; the genotyping technique was described in the previous publications [3].

The groups were genotyped by nucleotide sequence variants rs1799945 (H63D), rs1800562 (C282Y), rs1800730 (S65C) of *HFE* gene, rs113993960 (ΔF508) of *CFTR* gene, rs28929474 (PIZ), rs17580 (PIS) of *SERPINA1* gene by polymerase chain reaction followed by the analysis of fragment length polymorphism on polyacrylamide gels.

For genotyping by variant rs1799945 (H63D) we used the primers 5’-TGGTCTTTCCTTGTTTGAAGC-3’(F) and 5’-TCCATAATAGTCCAGAAGTCAACAG-3’(R). The mixture for PCR, 25 μl included: 75 mM Tris-HCI (pH 9.0), 20 mM (NH_4_)_2_SO_4_, 0.01% Tween-20, 2.5 mM MgCl_2_ by 0.4 mM of each primer, 0.2 mM of dNTP mixture, 2 μg DNA, 1 activity unit of DNA-polymerase. Amplifcation was performed under the following conditions: 35 cycles including denaturation — at 95°C for 30 s, annealing of primers — at 60°C for 30 s, and elongation — at 72°C for 30 s. Restriction was performed with 10 activity units of restrictase Ksp22 I (SibEnzyme, Russia). The amplifcation product size was 195 bps. After restriction in CC genotype there were detected the products 137 and 58 bps, in GG genotype — 195 bps, in heterozygous CG genotype — 195, 137, and 58 bps.

For genotyping by rs1800562 (C282Y) there were used the primers 5’-CCCTGGGGAAGAGCAGAGAT-3’(F) and 5’-CCTTTGATTGCCACCCTC-3’(R). The mixture for PCR, 25 μl included: 75 mM Tris-HCI (pH 9.0), 20 mM (NH_4_)_2_SO_4_, 0.01% Tween-20, 2.5 mM MgCl_2_ by 0.4 mM of each primer, 0.2 mM of dNTP mixture, 2 μg DNA, 1 activity unit of DNA-polymerase. Amplifcation was performed under the following conditions: 35 cycles including denaturation — at 95°C for 30 s, annealing of primers — at 60°C for 30 s, and elongation — at 72°C for 30 s. Restriction was performed with 10 activity units of restrictase Rsa I (SibEnzyme, Russia). The amplification product size was 207 bps. After restriction in AA genotype there were detected the products 137 and 76 bps, in GG genotype — 207 bps, in heterozygous GA genotype — 207, 131, and 76 bps.

For genotyping by rs1800730 (S65C) there were used the primers 5’-TGTTGCTCTGTCTCCAGGT-3’(F) and 5’-CTGGAAACCCATGGAGTTC-3’(R). The mixture for PCR, 25 μl included: 75 mM Tris-HCI (pH 9.0), 20 mM (NH_4_)_2_SO_4_, 0.01% Tween-20, 3.5 mM MgCl_2_ by 0.8 mM of each primer, 0.2 mM of dNTP mixture, 2 μg DNA, 1 activity unit of DNA-polymerase. Amplification was performed under the following conditions: 35 cycles including denaturation — at 95°C for 30 s, annealing of primers — at 58°C for 30 s, and elongation — at 72°C for 30 s. Restriction was performed with 10 activity units of restrictase Hinf I (SibEnzyme, Russia). The amplification product size was 171 bps. After restriction in TT genotype there were detected the products 171 bps, in AA genotype — 133 and 38 bps, in heterozygous AT genotype — 171, 133, and 38 bps.

For genotyping by rs113993960 (ΔF508) of *CFTR* gene there were used the primers 5’-TTTTCCTGGATTATGCCTGGCACC-3’(F) and 5’-GTTGGCATGCTTTGATGACGCTTC-3’(R). The mixture for PCR, 25 μl included: 75 mM Tris-HCI (pH 9.0), 20 mM (NH_4_)_2_SO_4_, 0.01% Tween-20, 2.5 mM MgCl_2_ by 0.4 mM of each primer, 0.2 mM of dNTP mixture, 2 μg DNA, 1 activity unit of DNA-polymerase. Amplification was performed under the following conditions: 33 cycles including denaturation — at 95°C for 30 s, annealing of primers — at 63°C for 30 s, and elongation — at 72°C for 30 s. Amplification products were detected in 10% polyacrylamide gel followed by ethidium bromide staining. The amplification product size in II genotype was 97 bps, in ID — 97, 94, and 3 bps, in DD — 94 and 3 bps.

For genotyping by rs28929474 (PIZ) we used the primers 5’-ATAAGGCTGTGCTGACCATCGTC-3’(F) and 5’-TTGGGTGGGATTCACCACTTTTC-3’(R). The mixture for PCR, 25 μl included: 75 mM Tris-HCI (pH 9.0), 20 mM (NH_4_)_2_SO_4_, 0.01% Tween-20, 2.5 mM MgCl_2_ by 0.4 mM of each primer, 0.2 mM of dNTP mixture, 2 μg DNA, 1 activity unit of DNA-polymerase. Amplification was performed under the following conditions: 35 cycles including denaturation — at 95°C for 30 s, annealing of primers — at 56°C for 30 s, and elongation — at 72°C for 30 s. Restriction was performed with 10 activity units of restrictase Taq I (SibEnzyme, Russia). The amplification product size was 179 bps. After restriction in GG genotype there were detected the products 160 and 19 bps, in AA genotype — 179 bps, in heterozygous GA genotype — all the mentioned products (179, 160, and 19 bps).

For genotyping by rs17580 (PIS) there were used the primers 5’-TGAGGGGAAACTACAGCACCTCG-3’(F) and 5’-AGGTGTGGGCAGCTTCTTGGTCA-3’(R). The mixture for PCR, 25 μl included: 75 mM Tris-HCI (pH 9.0), 20 mM (NH_4_)_2_SO_4_, 0.01% Tween-20, 3.5 mM MgCl_2_ by 0.8 mM of each primer, 0.2 mM of dNTP mixture, 2 μg DNA, 1 activity unit of DNA-polymerase. Amplification was performed under the following conditions: 35 cycles including denaturation — at 95°C for 30 s, annealing of primers — at 61°C for 30 s, and elongation — at 72°C for 30 s. Restriction was performed with 10 activity units of restrictase Taq I (SibEnzyme, Russia). The amplification product size was 121 bps. After restriction in AA genotype there were detected the products 100 and 21 bps, in TT genotype — 121 bps, in heterozygous AT genotype — all the mentioned products (121, 100, and 21 bps).

### Statistical analysis

The obtained genotyping findings were statistically processed using the software package SPSS 16.0. By means of criterion *χ*^2^ we assessed the compliance of genotype frequencies with Hardy-Weinberg equilibrium in a control group. The comparison of the groups by frequencies of genotypes and alleles, the relative risk by a certain allele or genotype were calculated using the contingency tables using Pearson coefficient *χ*^2^, Fisher’s exact paired test with Yates’ correction for continuity. The differences were considered significant if p<0.05.

## Results

**Figure 1.**
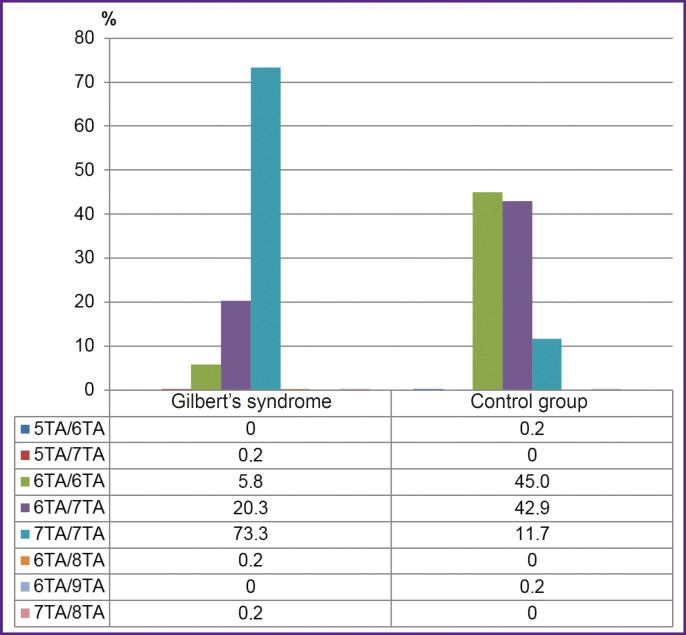
Genotype frequencies of rs3064744 mutations of *UGT1A1* gene in Gilbert’s syndrome group and the control group (p<0.001)

Figure 1 demonstrates the genotype frequencies of rs3064744 (the number of TA-repeats in a promoter) mutation of *UGT1A1* gene in GS group and the control group. By the frequencies of variant rs3064744 genotypes there were found significant differences between the groups (P<0.001). Genotype 7TA/7TA of rs3064744 mutation was more frequently found in subjects with unconjugated hyperbilirubinema compared to the controls (OR=20.6; 95% CI: 14.2-29.9; p<0.001).

In the control group the observed genotype frequencies of the nucleotide sequence variants rs1799945 (H63D), rs1800562 (C282Y), rs1800730 (S65C) of *HFE* gene, rs113993960 (ΔF508) of *CFTR* gene, rs28929474 (PIZ), rs17580 (PIS) of *SERPINA1* gene corresponded to those expected according to Hardy-Weinberg equilibrium (x^2^=0.60, 0.38, 0.16, 0.001, 0.05, 0.03, respectively).

**Figure 2.**
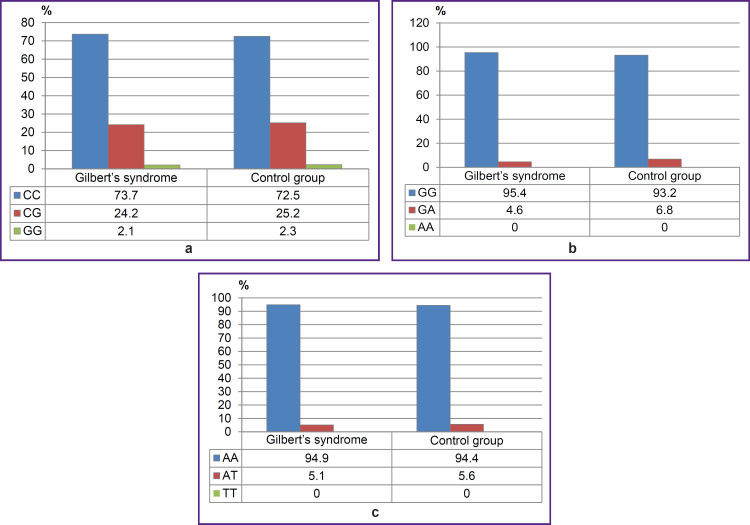
Genotype frequencies of the variants rs1799945 (H63D) (a), s1800562 (C282Y) (b), and rs1800730 (S65C) (c) of *HFE* gene in Gilbert’s syndrome group and the control group

**Figure 3.**
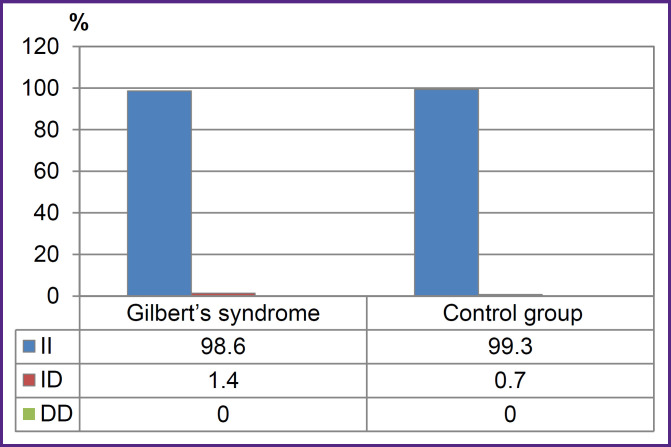
Genotype frequencies of the variants rs113993960 (ΔF508) of *CFTR* gene in Gilbert’s syndrome group and the control group

**Figure 4.**
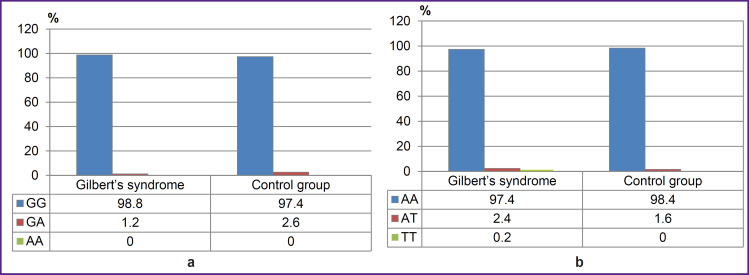
Genotype frequencies of the variants rs28929474 (PIZ) (a), rs17580 (PIS) (b) of *SERPINA1* gene in Gilbert’s syndrome group and the control group

There were found no significant differences between GS group and the control group by the frequencies of genotypes and alleles rs1799945 (H63D), rs1800562 (C282Y), rs1800730 (S65C) of *HFE* gene, rs113993960 (ΔF508) of *CFTR* gene, rs28929474 (PIZ), rs17580 (PIS) of *SERPINA1* gene (p>0.05). Figures 2-4 represent the frequencies of thee genotypes.

64% of group GS participants appeared not to be the carriers of rare alleles of the studied variants of oligogenic hepatic diseases — rs1799945 (H63D), rs1800562 (C282Y), rs1800730 (S65C) of *HFE* gene, ΔF508 of *CFTR* gene, rs28929474 (PIZ), rs17580 (PIS) of *SERPINA1* gene.

6 of 24 GS group patients with genotype 6TA/6TA of variant rs3064744 were the carriers of heterozygous CG genotype rs1799945 (H63D) of *HFE* gene, 1 patient — heterozygous GA genotype rs28929474 (PIZ) of *SERPINA1* gene, as well as 1 patient *—* heterozygous AT genotypes rs17580 (PIS) of *SERPINA1* gene and ID rs113993960 (ΔF508) of *CFTR* gene.

32 of 84 subjects with 6TA/7TA genotype of rs3064744 variants were the carriers of one major mutation in heterozygous state — rs1800730 (S65C) of *HFE* gene/ rs113993960 (ΔF508) of *CFTR* gene/rs28929474 (PIZ) of *SERPINA1* gene, or in homozygous/heterozygous state — rs1799945 (H63D) of *HFE* gene.

102 of 303 subjects with 7TA/7TA genotype rs3064744 were found to have the carriership of rare alleles of variants rs1799945 (H63D), rs1800562 (C282Y), rs1800730 (S65C) of *HFE* gene, rs113993960 (ΔF508) of *CFTR* gene, rs28929474 (PIZ), rs17580 (PIS) of *SERPINA1* gene. So, a 14-year-old boy (7TA/7TA rs3064744; total bilirubin concentration: 57.0 μmol/L that came in view of the doctor, who referred the patient for the examination) was a carrier of heterozygous CG genotype rs1799945 (H63D) of *HFE* gene, and heterozygous AT genotype rs17580 (PIS) of *SERPINA1* gene. 2 subjects with 7TA/7TA genotype rs3064744 and heterozygous CG genotype rs1799945 (H63D) of *HFE* gene also had heterozygous AT genotype rs1800730 (S65C) of *HFE* gene (a 39-year-old woman with total bilirubin concentration 51.8 μmol/L, and a 46-year-old man with no biochemical blood count). A 17-year-old girl (7TA/7TA rs3064744) was found to have a heterozygous AT genotype rs1800730 (S65C) of *HFE* gene and heterozygous ID genotype ΔF508 of *CFTR* gene (total bilirubin concentration — 39.4 μmol/L).

In a 22-year-old girl with an episode of high concentration of total (170.0 μmol/L) and unconjugated bilirubin (155.8 μmol/L) there was verified a rare genotype 5TA/7TA of the variant rs3064744 of *UGT1A1* gene, heterozygous genotype CG of the variant rs1799945 (H63D) of *HFE* gene and homozygous genotype TT of the variant rs17580 (PIS) of *SERPINA1* gene. Moreover, according to Sanger sequencing of *UGT1A1* gene, the patient was a carrier of missensevariant rs2125984650 in the frst exon of *UGT1A1* gene (c.188A>T, p.Asp63Val) of uncertain clinical significance.

67.8% patients in the control group were not the carriers of rare alleles of the studied variants rs1799945 (H63D), rs1800562 (C282Y), rs1800730 (S65C) of *HFE* gene, rs113993960 (ΔF508) of *CFTR* gene, rs28929474 (PIZ), rs17580 (PIS) of *SERPINA1* gene. This group already had the carriers of rare alleles of several variants; however, due to the lack of data on hepatobiliary system condition, total and unconjugated bilirubin concentration, the description of these combinations holds no scientific value.

There were found no significant differences between the groups by the genotype combinations of variants rs3064744 of *UGT1A1* gene with the genotypes of rs1799945 (H63D), rs1800562 (C282Y), rs1800730 (S65C) variants of *HFE* gene, rs113993960 (ΔF508) of *CFTR* gene, rs28929474 (PIZ), rs17580 (PIS) of *SERPINA1* gene.

## Discussion

*HFE* gene (homeostatic iron regulator, 6p22.2) encodes the membrane protein, which is believed to regulate iron absorption due to its effect on the interaction of transferrin and its receptor [4]. *HFE* gene defects (rs1799945 — g.26090951C>G, p.His63Asp, H63D; rs1800562 — g.26092913G>A, p.Cys282Tyr, C282Y; rs1800730 — g.26090957A>T, p.Ser65Cys, S65C) result in hereditary hemochromatosis development (autosomal recessive inheritance), which is characterized by excessive iron absorption that in its turn leads to body intoxication, hepatic cirrhosis, and death [5].

*CFTR* gene (CF transmembrane conductance regulator, 7q31.2) encodes the protein, which functions as a chloride canal, controls ion and water absorption by epithelial cells. The most frequent gene mutation — ΔF508 (rs113993960, p. Phe508del) leads to impaired protein assembly and transport causing cystic fibrosis (autosomal recessive inheritance) [6]. Cystic fibrosis is characterized by chronic pulmonary infection and infammation, pancreatic exocrine insufficiency, male infertility, as well as can cause several accompanying diseases related to cystic fibrosis, such as diabetes and hepatic pathology [7]. Heterozygous ΔF508 mutation carriership is associated with a high risk of developing pancreatitis, male infertility, bronchiectases, diabetes, cholelithiasis, and other conditions [8].

*SERPINA1* gene (serpin family A member 1, 14q32.13) encodes the protein — alpha-1-antitrypsin, which is synthesized in the liver, bone marrow, lymphoid tissue, intestine; the gene defects result in alpha-1-antitrypsin deficiency (autosomal recessive inheritance) expressed in chronic obstructive pulmonary diseases, emphysema, chronic hepatic diseases [9]. Most frequent variants of *SERPINA1* gene leading to alpha-1-antitrypsin deficiency — rs28929474 (c.1096G>A, p.Glu366Lys, PIZ), rs17580 (c.863A>T, p.Glu288Val, PIS).

The present work studied the relationship of proteins encoded by *HFE, CFTR, SERPINA1, UGT1A1* genes using STRING platform (https://string-db.org). According to the platform data, the study using affinity chromatography indicates possible functional relationship between protein products of *CFTR* and *SERPINA1* genes. The co-expression of orthologs of *HFE* and *SERPINA1* genes was observed in brown rats and Oryza Sativa.

Gene relationship is known to alter gene expression, have an effect on the manifestation of clinical symptoms. Some diseases previously believed as multi-genic (one gene — one disease) now are referred to oligigenic, or even polygenic due to the effect of environmental factors on their development [10]. At present, according to the OLIgogenic diseases DAtabase (OLIDA; https://olida.ibsquare.be/), cystic fibrosis and hereditary hemochromatosis can be referred to oligogenic conditions, their development is contributed by *CFTR* and *HFE*, genes, and some others.

In literature there are reports on the combined carriership of *CFTR* and *SERPINA1* gene variants in case of a clinical picture of cystic fibrosis, though without homozygous carriership of *CFTR* gene variant. According to Ramos et al. [11], it gives the evidence that such phenotype results from the combination of genotypes of several genes (the study in addition to *SERPINA1* gene included *SCNN1A, SCNN1B, SCNN1G* genes). The effect of alpha-1-antitrypsin on CFTR protein level was shown in the study on cell culture [12]. There are the data *HFE* gene mutations are related to the development of meconium ileus and hepatic pathology in cystic fibrosis [13].

Taking in consideration the above mentioned, we can suppose that GS course can be also influenced by *HFE, CFTR* И *SERPINA1* gene variants. According to the study findings, there were revealed no association of GS with nucleotide sequence variants rs1799945 (H63D), rs1800562 (C282Y), rs1800730 (S65C) of *HFE* gene, ΔF508 of *CFTR* gene, rs28929474 (PIZ), rs17580 (PIS) of *SERPINA1* gene. However, some patients were found to have the combined carriership of genotype 6TA/6TA or 6TA/7TA rs3064744 and rare alleles of several variants from those under study. Moreover, some patients with genotype 7TA/7TA rs3064744 were also revealed to have the combinations with other variants, which can explain significant rise in bilirubin concentration.

Thus, in case the individuals with GS phenotype (when excluding other reasons of unconjugated hyperbilirubinemia except genetic ones) have the significant increase in total/unconjugated bilirubin concentration or/and in genotype 6TA/6TA, 6TA/7TA rs3064744 — it is reasonable to perform a research to search for the carriership of rare alleles of rs1799945 (H63D), rs1800562 (C282Y), rs1800730 (S65C) variants of *HFE* gene, rs113993960 (ΔF508) variants of *CFTR* gene, rs28929474 (PIZ), rs17580 (PIS) variants of *SERPINA1* gene.

**The present study was limited** by the lack of information on a clinical course of unconjugated hypebilirubinemia in GS group (available are the data on their total and unconjugated bilirubin level that made the patient to consult a doctor). The control group was a random sampling that does not preclude the presence of some individuals with diagnosed or non-diagnosed GS.

## Conclusion

The present study revealed no significant differences in the frequencies of genotypes and alleles of nucleotide sequence variants rs1799945 (H63D), rs1800562 (C282Y), rs1800730 (S65C) of *HFE* gene, rs113993960 (ΔF508) of *CFTR* gene, rs28929474 (PIZ), rs17580 (PIS) of *SERPINA1* gene between the patients with unconjugated hyperbilirubinemia and the controls. The analysis of the combined genotypes of rs3064744 variant (the number of TA repeats in a pronotor) of *UGT1*A1 gene with the genotypes of rs1799945 (H63D), rs1800562 (C282Y), rs1800730 (S65C) variants of *HFE* gene, rs113993960 (ΔF508) of *CFTR* gene, rs28929474 (PIZ), rs17580 (PIS) of *RPINA1* gene also revealed no significant differences between the groups. Thus, it may be concluded that the sampling under study had nucleotide sequence variants rs1799945 (H63D), rs1800562 (C282Y), rs1800730 (S65C) of *HFE* gene, rs113993960 (ΔF508) of *CFTR* gene, rs28929474 (PIZ), rs17580 (PIS) of *SERPINA1* gene, non-associated with unconjugated hypebilirubinemia.
